# The role of the outer setting in implementation: associations between state demographic, fiscal, and policy factors and use of evidence-based treatments in mental healthcare

**DOI:** 10.1186/s13012-019-0944-9

**Published:** 2019-11-13

**Authors:** Eric J. Bruns, Elizabeth M. Parker, Spencer Hensley, Michael D. Pullmann, Philip H. Benjamin, Aaron R. Lyon, Kimberly E. Hoagwood

**Affiliations:** 1 University of Washington, 6200 NE 74th Street, Building 29, Suite 110, Seattle, WA 98115 USA; 2 New York University, One Park Avenue at East 33rd, 7-310, New York, NY 10016 USA

**Keywords:** Mental health, Evidence-based treatments, Public mental health systems, State policy, Outer setting, Implementation research, Implementation strategies

## Abstract

**Background:**

Despite consistent recognition of their influence, empirical study of how outer setting factors (e.g., policies, financing, stakeholder relationships) influence public systems’ investment in and adoption of evidence-based treatment (EBT) is limited. This study examined associations among unmodifiable (e.g., demographic, economic, political, structural factors) and modifiable (e.g., allocation of resources, social processes, policies, and regulations) outer setting factors and adoption of behavioral health EBT by US states.

**Methods:**

Multilevel models examined relationships between state characteristics, an array of funding and policy variables, and state adoption of behavioral health EBTs for adults and children across years 2002–2012, using data from the National Association for State Mental Health Program Directors Research Institute and other sources.

**Results:**

Several *unmodifiable* state factors, including per capita income, controlling political party, and Medicaid expansion, predicted level of state *fiscal investments* in EBT. By contrast, *modifiable* factors, such as interagency collaboration and investment in research centers, were more predictive of state *policies* supportive of EBT. Interestingly, level of adult EBT adoption was associated with state fiscal supports for EBT, while child EBT adoption was predicted more by supportive policies. State per capita debt and direct state operation of services (versus contracting for services) predicted both child and adult EBT adoption.

**Conclusions:**

State-level EBT adoption and associated implementation support is associated with an interpretable array of policy, financing, and oversight factors. Such information expands our knowledge base of the role of the outer setting in implementation and may provide insight into how best to focus efforts to promote EBT for behavioral health disorders.

Contributions to the literature

- Implementation-oriented policy research is scarce. The current paper uses data from a longstanding federal effort to track implementation of evidence-based treatments (EBTs) in US states to evaluate the influence of outer setting factors on EBT investment, support, and adoption.
- The study found “unmodifiable” factors (e.g., per capita income, controlling political party) predicted *funding support* for EBTs, while “modifiable” factors (e.g., collaboration, investment in research) predicted presence of EBT-supportive state *policies*.
- The study also found that different types of outer setting factors were associated with adoption of adult EBTs versus child EBTs.
- The paper expands knowledge of the role of the outer setting in implementation and provides insights into how best to focus state-level efforts to promote EBT.

## Background

Evidence-based treatment (EBT) has become a standard of care in mental health service delivery [1, 2], while also holding promise for improving population health [3–5]. A legion of barriers to translating research into real-world practice, however, have been identified, including economic constraints, lack of political will, scientific uncertainty, and institutional culture and structure [6, 7]. Among these barriers, theory [8, 9], research [10], and “on the ground” experience [11, 12] all tell us that policy, fiscal, economic, social, and other factors in the “outer setting” of the implementation ecology will greatly influence the degree to which EBT will be available in a system and aid individuals in need.

In the US, states are in a clear position to influence the degree to which behavioral health services and systems invest in research and EBT [13–15]. Legislators, state health and behavioral health leaders, and other policy-makers allocate resources for EBT and supportive technologies and infrastructure; determine covered services (such as in state Medicaid plans in the USA); and enact regulations that support availability and implementation of EBT. Overall, however, empirical study of state support to EBT adoption and investment is scant [16, 17], as is research on outer setting determinants, when compared to mechanisms operating at other levels [18].

### Existing knowledge base

In previous research [19], we used data from regular surveys of US state mental health authority (SMHA) program directors (individuals charged with overseeing mental health policy, fiscal, workforce, and other initiatives at a statewide level), to examine state trends in EBT adoption and penetration rates and EBT implementation supports by states from 2001 to 2012. While results found moderate levels of EBT adoption for three child-focused EBTs (25–50% of the states reported adopting each of these) and three adult-focused EBTs (65%–75% of the states reported adopting each of these), these services did not reach the vast majority of those in need. There were very low penetration rates (0.75–2.5% of those in need) as well as trends indicating decreasing levels of fiscal support for EBT. States varied greatly, however, across reported rates of EBT funding, supportive policies, and EBT adoption.

Health policy scholars have proposed an array of factors in the fiscal and policy context (i.e., outer setting) that may influence uptake of EBT [9, 20]. Outer setting variables are notoriously important for implementation, but simultaneously difficult to evaluate and influence. For instance, the Society for Implementation Research Collaboration’s (SIRC) comprehensive Instrument Review Project identified only four instruments that capture outer setting processes [21]. Efforts to operationalize these variables and their taxonomies have also been scant. For example, initial versions of the widely applied Consolidated Framework for Implementation Research [9] characterized “external policies and incentives” as a “broad construct” within the outer setting domain, but dedicated only a single sentence to its definition.

Other, more recent, theories, such as the Exploration, Preparation, Implementation, and Sustainment (EPIS) framework [8], more explicitly identify factors within the outer context (e.g., leadership, funding/contracting, inter-organizational networks) that are proposed to be influential across phases of implementation, as well as “bridging factors” that may link the outer context to influential factors at the organizational (or inner setting) level. Other theories characterize factors in the outer setting as including both “unmodifiable” (e.g., economic, political) versus “modifiable” (e.g., funding, regulations, networks) factors [22], potentially providing guidance around how these theories might be applied to action. To date, however, there has been little robust application of such theoretical drivers to implementation research or “on the ground” use [18].

Nonetheless, a small number of previous studies (mostly that predate the publication of the CFIR and/or the first volume of *Implementation Science*) have attempted to identify and characterize the types of strategies used by states to promote EBT [23]. Other scholars have used an expert consensus process to identify a comprehensive array of potential outer setting implementation strategies [24]. No previous empirical studies, however, have asked what characteristics of states or state behavioral health systems are associated with EBT adoption or investment. Such a study could inform the emerging field of EBT dissemination and implementation policy [16] as well as provide insight into how best to focus efforts to promote EBT in public systems.

### Current aims

**Fig. 1:**
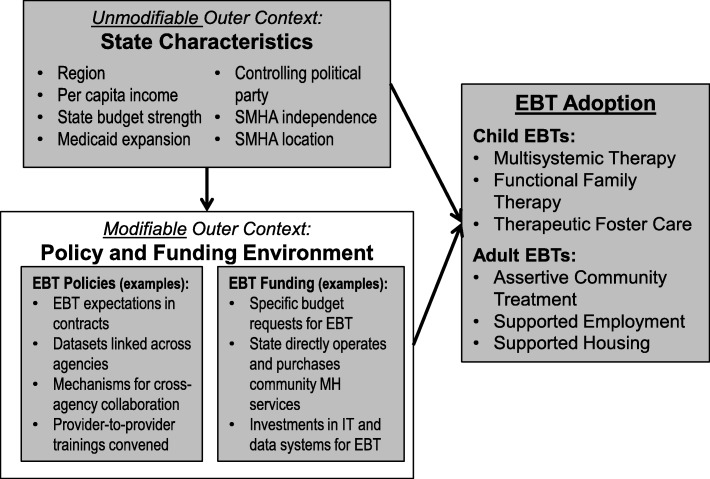
Conceptual framework of associations among state characteristics and evidence-based treatment policies, funding, and adoption

This article presents the results of a study to help fill this knowledge gap, using longitudinal survey data of SMHA directors from all 50 US states and territories compiled by the NASMHPD Research Institute (NRI) and other data sources to explore the strengths of relationships between state factors and state activities and the dependent variables of EBT investments and adoption. As a first step, we developed a conceptual framework for the study (Fig. 1) that organized data relevant to state efforts to promote EBT adoption (“modifiable” external factors, mostly from NRI surveys) as well as state data from other sources on factors proposed by previous theory and research [9, 20, 22] to be influential (“unmodifiable” factors).

As depicted in Fig. 1, we hypothesized that certain unmodifiable state factors would be associated with the degree to which policy-makers modify the implementation ecology for EBT (e.g., through fiscal investments and supportive policies). We also hypothesized that both modifiable state factors (e.g., state fiscal and policy supports) and unmodifiable factors (e.g., state spending patterns and party in political control) would be associated with degree of EBT adoption. Importantly, because of the lack of previous research on this topic, and our use of secondary data, we did not hypothesize specific variables within these major domains that would emerge as significant predictors.

The study sought to answer two broad research questions that extend from this conceptual framework. First, what *unmodifiable* state factors (e.g., economic, geographic, political, structural) are associated with state-level EBT financial investment and supportive policies? Second, what *unmodifiable* (e.g., economic, geographic, political, structural) and *modifiable* (i.e., SMHA structures, social processes, EBT-related investments and policies) factors are associated with actual state-level adoption of six leading behavioral health EBTs (three focused on adults and three focused on children)?

## Methods

### Measures and data sources

The current study was conducted in the USA. Data originated from five publically available sources. The count of adults and children in each state and the per capita income of each state come from the US Census Bureau [25] and US Department of Commerce [26], respectively. Each state’s status with respect to Medicaid expansion under the Affordable Care Act was obtained from publically available data from the Kaiser Family Foundation [27]. Each state’s budget surplus or deficit as well as controlling political party come from Harvard University’s public online “Dataverse” [28]. For controlling political party, we calculated a “controlling party index” based on which party controlled the two houses of the legislature and the executive (Governor) branch (Republican control = 0; Democratic = 1). Thus, this variable could range from 0 (all three branches Republican) to 3 (all three branches Democratic).

All other variables come from the State Profiles System (SPS) and Uniform Reporting System (URS), datasets made available by NRI. The SPS is administered to representatives of SMHAs and asks a wide range of questions about the structure and functioning of the SMHA. The URS is a reporting system as part of SAMHSA’s Community Mental Health Block Grant (CMHBG). It compiles numbers and characteristics of clients served by the each state. It also asks about the use of six EBTs identified in the early 2000s by SAMHSA as being particularly relevant to the populations of focus of the CMHB—adults with serious mental illness (SMI) and children with serious emotional and behavioral disorders (SEBD). Additional details can be found from recent reports [13, 15]. We used 34 items from these NRI datasets: 6 related to the provision of specific EBTs, 11 related to “state characteristics,” 12 about policy supports to promote EBT adoption, and 5 about fiscal supports to EBT adoption. A complete list of items included in analyses can be found in Table 1. Below, we provide more details on variables from the SPS and URS.

**Table 1:** Summary of variables included in analyses

Item	Source
Provision of specific EBTs (yes/no)	
Multisystemic therapy	Uniform Reporting System
Therapeutic foster care	Uniform Reporting System
Functional family therapy	Uniform Reporting System
Supported employment	Uniform Reporting System
Supported housing	Uniform Reporting System
Assertive community treatment	Uniform Reporting System
State characteristics	
Geographic region (South, West, Midwest, Northeast)	US Census Bureau
Number of adults and children	US Census Bureau
Per capita income	US Department of Commerce
Amount of budget surplus or deficit	Harvard Dataverse
Controlling political party	Harvard Dataverse
Medicaid expansion status (yes/no)	Kaiser Family Foundation
Controlled per capita expenditures of the SMHA (in millions)	State Profiles Survey
SMHA directly operates community-based mental health programs or funds county or city mental health authorities which, in turn, fund local provider agencies to provide care (yes/no)	State Profiles Survey
SMHA located within another state agency (yes/no)	State Profiles Survey
SMHA director sit as a member of the Governor’s cabinet (yes/no)	State Profiles Survey
Research is located within the SMHA (yes/no)	State Profiles Survey
Research is located outside of the SMHA (yes/no)	State Profiles Survey
SMHA promotes consumer/survivor participation in resource allocations at the SMHA level (yes/no)	State Profiles Survey
SMHA has initiatives underway to work with other state government agencies to coordinate, reduce, or eliminate barriers between delivery systems and funding streams to the provision of appropriate mental health services (yes/no)	State Profiles Survey
Representatives from other state government agencies participate as members of SMHA’s mental health planning Council/Group (yes/no)	State Profiles Survey
Information functions located inside of SMHA (yes/no)	State Profiles Survey
Information functions located outside of SMHA (yes/no)	State Profiles Survey
SMHA is involved in activities to downsize, reconfigure, close and/or consolidate one or more State mental hospitals (yes/no)	State Profiles Survey
Policy supports to promote EBT adoption	
Modification of information systems and data reports is used to promote the adoption of EBPs (yes/no)	State Profiles Survey
Monitoring of fidelity is used to promote the adoption of EBPs (yes/no)	State Profiles Survey
SMHA implemented or is it implementing a statewide client outcome monitoring system (yes/no)	State Profiles Survey
SMHA conduct research/evaluations on client change in functioning (yes/no)	State Profiles Survey
Internal staff are used to provide on-going training to providers related to evidence-based practices (yes/no)	State Profiles Survey
Expert consultants used to provide on-going training to providers related to evidence-based practices (yes/no)	State Profiles Survey
Collaboration with universities used to provide on-going training to providers related to evidence-based practices (yes/no)	State Profiles Survey
Establishment of research/training institute(s) is used to provide on-going training to providers related the evidence-based services (yes/no)	State Profiles Survey
Awareness/training is used to promote the adoption of EBPs (yes/no)	State Profiles Survey
Financial incentives are used to promote the adoption of EBPs (yes/no)	State Profiles Survey
Specific budget requests are used to promote the adoption of EBPs (yes/no)	State Profiles Survey
SMHA funds a research center/institute (yes/no)	State Profiles Survey
Fiscal supports to EBT adoption	
Incorporation in contracts is used to promote the adoption of EBPs (yes/no)	State Profiles Survey
SMHA integrated/linked/matched its client datasets with client datasets from any other agencies (yes/no)	State Profiles Survey
SMHA have initiatives underway to work with other state government agencies to coordinate, reduce, or eliminate barriers between delivery systems and funding streams to the provision of appropriate mental health services (yes/no)	State Profiles Survey
Internal staff used to provide on-going training to providers related to evidence-based practices (yes/no)	State Profiles Survey
Provider-to-provider training used to provide on-going training to providers related to evidence-based practices (yes/no)	State Profiles Survey
SMHA worked with academia in curriculum development to reflect evidence-based practices, promising practices, or value-based practices (yes/no)	State Profiles Survey

#### State characteristics

In addition to data from the Census Bureau, Department of Commerce, Kaiser Family Foundation, and Harvard Dataverse, we relied on 11 items from the SPS related to structural characteristics of each state and SMHA (see Table 1). These items ask whether the SMHA, for example, directly operates community-based mental health programs (versus contracts with others to provide care); is located within another state agency; includes representatives from other state government agencies on its mental health council; houses a research institute (within or outside of the SMHA); and promotes consumer participation in resource allocations.

#### EBT adoption

State-level adoption of behavioral health EBTs included six EBTs tracked by the NRI surveys over the study period. EBTs included three interventions for children with SEBD: therapeutic foster care [29, 30], multisystemic therapy [31, 32], and functional family therapy [33]; and three treatments for adults with SMI: supported housing [34], supported employment [35], and assertive community treatment [36, 37]. Because the NRI survey was intended to provide federal funding agencies with information on populations of specific interest (i.e., to the SAMHSA CMHBG), all of these interventions are intended for individuals with complex needs, and most are multimodal (i.e., include multiple strategies that address a range of factors that may influence individual and contextual needs).

SHMA representatives were asked about the availability of these six EBTs and had several response options that were dichotomized into 0 “EBT was not available” (not implementing, planning to implement) and 1 “EBT was available” (implementing in parts of the state, implementing statewide). An EBT availability index variable was created by calculating the amount of child and adult EBTs reported as available. Scores ranged from 0 (no EBTs available) to 10 (all 6 EBTs available). We did this for this index and several described below in order to aid interpretability and place several of the measures on similar scales. Data on EBT availability was limited to the years 2007 through 2012, when the URS was implemented as a state-level accountability mechanism by the Substance Abuse and Mental Health Services Administration.

#### State fiscal supports to promote EBT adoption

State EBT fiscal supports were assessed using 11 items from the SPS (see Table 1). SMHA representatives were asked to respond yes (coded as 1) or no (coded as 0) to statements such as “Specific budget requests are used to promote the adoption of EBTs,” “Financial incentives are used to promote the adoption of EBTs,” and “Modification of information systems and data reports is used to promote the adoption of EBTs.” An EBT investment index variable was created by calculating the percent of items related to EBT investments that were endorsed. Scores ranged from 0 (no items endorsed) to 10 (all items endorsed).

#### State policy supports to promote EBT adoption

State EBT policy supports were assessed using six items from the SPS (see Table 1). SMHA representatives were asked questions such as “Is incorporation in contracts used to promote adoption of EBTs,” and “Does your SMHA have initiatives underway to work with other state government agencies to coordinate, reduce, or eliminate barriers between delivery systems and funding streams to the provision of appropriate mental health services.” Response options were yes (coded as 1) or no (coded as 0). An EBT policy index variable was created by calculating the percent of items related to EBT policies that were endorsed. Scores ranged from 0 (no items endorsed) to 10 (all items endorsed).

### Years examined

SPS and URS data were collected for most variables in 2002, 2004, 2007, 2009, 2010, and 2012. Data on the provision of EBTs, however, were only available from 2007 to 2012.

### Sample

Data cover all 50 states and the District of Columbia. SPS data were available for most states in most years; response rates vary from a low of 84% (43 of 51) in 2005 to a high of 98% in 2009 and 2010 (50 out of 51). The average response rate across all 7 years was 92%. Data from the URS were available from every state from 2007 to 2012. Data from all other sources were complete.

### Data analysis

Descriptive statistics were run using STATA 13.1 [38]. Two-level hierarchical models were conducted using HLM 7.0 [39] to account for the nested structure of the data, where time and year were nested within state. We ran individual models using each individual predictor variable and linear time, as our sample size did not provide the power for multivariable model building or testing curvilinear time trends. Normal distributions were used for continuous outcome variables, and Bernoulli distributions were used for dichotomous outcome variables (availability or unavailability of individual EBTs within the state). Models were run using full maximum likelihood (FML) estimation due to unbalanced data timepoints within states. Randomly varying intercepts were permitted but slopes were fixed due to failure to achieve model convergence as a result of low power (only 50 states) and small variance (there was limited within-state change over time for most variables). Essentially, this resulted in analyses similar to repeated-measures ANOVAs with random effects for state intercept but not slope [40], using FML estimation to permit the inclusion of states with missing data points.

All models were adjusted for time centered at 2002, except those for the outcome EBT availability index. These models were adjusted for time centered at 2007 because data on EBT availability was limited to the years 2007, 2009, 2010, and 2012. We report the most basic models that only include the predictor and time variables. Because only 4 years of data were available for models examining EBT availability, the total possible sample size was *n* = 200; actual sample size for most models when including missing data points ranged from *n* = 135–152; budget surplus and per capita expenditures were only asked in three of those years (*n* = 99). For all other models, with six possible years of data, total possible sample size was *n* = 300, and actual sample sizes ranged from *n* = 258–270.

Because the locus of analysis is the universe of all 50 US states (and response rates were close to 100% across years (M = 92%)), rather than a sample of some greater population of states, sampling error is of limited concern. We capitalize on the repeated measures nature of the study (e.g., longitudinally collected variables, for which change over time is not of interest) to provide some statistical accounting for measurement error. Therefore, significance testing was undertaken primarily as a means of flagging meaningful associations. Due to the exploratory nature of the study, we flagged and discuss as meaningful all associations with a significance level of *p* < 0.1.

## Results

Both EBT indices approximated normality; the mean EBT investment index score across all years was 5.6 (SD = 2.1, skewness = − 0.12, kurtosis = − 0.26) and the mean EBT policies index score across all years was 6.4 (SD = 2.6, skewness = − 0.36, kurtosis = − 0.50).

### Associations between state characteristics and EBT fiscal and policy supports

#### State characteristics and policy supports to promote EBT adoption

Table 2 displays the MLM estimates for the models exploring the associations between state characteristics and policy supports to promote EBT adoption. For states where the SMHA collaborates with other agencies, the EBT policy index increased by 2.83 points (*p* < 0.001). For states where representatives from state government agencies are members of the SMHA planning group, the EBT policy index increased by 1.74 points (*p* = 0.001). For states where research is conducted within the SHMA, the EBT policy index increased by 0.87 points (*p* = 0.016); and for states where research is conducted outside the SHMA, the EBT policy index increased by 0.85 points (*p* = 0.03). For states where the SMHA is located within another state agency, the EBT policy index decreased by 0.74 points (*p* = 0.088). All other state characteristics examined were not significantly associated with the EBT policy index.

**Table 2:** Associations between state characteristics and state EBT fiscal and policy supports and EBT availability

	Outcome 1: Fiscal supports to promote EBT adoption		Outcome 2: Policy supports to promote EBT adoption		Outcome 3: EBT availability	
Parameter	Unstandardized coef ^a^	SE	Unstandardized coef ^a^	SE	Unstandardized coef^b^	SE
Political party in control	0.260^	0.141	0.248	0.177	− 0.148	0.191
Budget surplus (*z* score)	− 0.088	0.096	0.123	0.146	− 0.750***	0.204
Per capita income (*z* score)	0.433^	0.253	0.307	0.225	− 0.036	0.236
Region						
South	− 0.00039	0.271	0.211	0.288	0.501	0.449
West	− 0.594	0.336	− 0.513	0.343	0.355	0.560
Midwest	− 0.126	0.334	− 0.140	0.370	− 0.937*	0.340
Northeast	0.721	0.357	0.443	0.433	0.081	0.601
2013 Medicaid expansion	0.950*	0.372	− 0.109	0.432	0.445	0.655
SMHA funds county (single or multicounty) or city mental health authorities which, in turn, fund local provider agencies or directly provide mental health services						
Directly operates community-based programs	− 0.241	0.507	0.049	0.666	1.037*	0.526
Funds county or city MH authorities	− 0.039	0.434	0.047	0.421	0.139	0.465
Directly funds, but does not operate community-based agencies	Ref		Ref		Ref	
SMHA is located within another state agency (ref = SMHA is an independent department/agency)	− 0.511	0.427	− 0.743^	0.434	− 0.060	0.690
SMHA Director sits as a member of the Governor’s cabinet (ref = no)	0.025	0.355	0.177	0.408	− 0.474	0.426
Research located within the SMHA (ref = no)	0.822*	0.372	0.870*	0.357	0.228	0.484
Research located outside the SMHA (ref = no)	0.906**	0.342	0.855*	0.389	0.338	0.619
SMHA promotes consumer/survivor participation in resource allocations at the SMHA level (ref = no)	0.185	0.392	0.095	0.470	0.151	0.099
SMHA has initiatives underway to work with other state government agencies to coordinate, reduce, or eliminate barriers between delivery systems and funding streams to the provision of appropriate mental health services (ref = no)	0.515*	0.246	2.836***	0.317	0.359	0.430
Representatives from other state government agencies participate as members of your SMHA’s mental health planning Council/Group (ref = no)	0.953	0.600	1.739**	0.532	0.112	0.466
Information management functions located within SMHA (ref = no)	− 0.168	0.299	− 0.187	0.421	No data available	
Information management functions located outside SMHA (ref = no)	− 0.470	0.622	− 0.853	0.579	No data available	
SMHA currently involved in activities to downsize, reconfigure, close and/or consolidate one or more state mental hospitals (ref = no)	− 0.033	0.228	0.416	0.301	− 0.028	0.387
Per capita expenditures (*z* score)	− 0.054	0.183	0.209	0.193	− 0.055	0.180
Controlled per capita expenditures for SMHA (*z* score)	0.038	0.325	0.256	0.258	− 0.071	0.215
Per capita mental health expenditures (*z* score)	0.274	0.196	0.277	0.259	0.097	0.271
Count of number of available child EBTs	0.238^	0.138	0.521**	0.185		
Count of number of available adult EBTs	0.286*	0.115	0.103	0.181		
Count of number of available EBTs (child and adult)	0.204*	0.081	0.242*	0.119		

*EBT* evidence-based treatment

^a^Adjusted for linear time centered at 2002

^b^Adjusted for linear time centered at 2007

^*p* < 0.10, * *p* < 0.05, ** *p* < 0.01, *** *p* < 0.001

#### State characteristics and fiscal supports to promote EBT adoption

Table 2 displays the MLM estimates for the models exploring the associations between state characteristics and fiscal supports to promote EBT adoption. For states where research is conducted outside the SMHA, the EBT investment index increased by 0.91 points (*p* = 0.009); and where research is conducted inside the SMHA, the EBT investment index increased by 0.82 points (*p* = 0.029). In states where Medicaid was expanded in 2013, the EBT investment index increased 0.95 points (*p* = 0.011). For states where the SMHA collaborates with other agencies, the EBT investment index increased by 0.51 points (*p* = 0.038). For each point on the four-point “controlling party index” (increasing Democratic control), the EBT investment index increased by 0.26 points (*p* = 0.066). State per capita income was also associated with the EBT investment index (*p* = 0.089). All other state characteristics examined were not significantly associated with the EBT investment index.

### Associations between state characteristics and availability of EBTs

#### State characteristics and the availability of EBTs

Table 2 displays the MLM estimates for the models exploring the associations between state characteristics and EBT availability at the state level. For every one standard deviation change in the state’s budget surplus, the EBT availability index decreased by 0.75 points (*p* < 0.001). For states located in the Midwestern part of the country, the EBT availability index deviated from the grand mean by 0.94 points (*p* = 0.02). For states where the SMHA directly operates community-based programs, versus directly funds but does not operate community-based programs, the EBT availability index increased by 1.03 points (*p* = 0.051). All other state characteristics examined were not significantly associated with the EBT availability index.

#### Fiscal supports and specific EBT availability

Table 3 displays the MLM estimates for the models exploring the associations between fiscal supports to promote EBT adoption and the availability of adult and child EBTs. Each 1 point increase in the EBT investment index was associated with a 30% increase in the odds a state will have SE available (OR = 1.30, *p* < 0.001). Each 1 point increase in the EBT investment index was associated with a 13% increase in the odds a state will have FFT available (OR = 1.13, *p* = 0.044). Each 1 point increase in the EBT investment index was associated with a 9% increase in the odds a state will have SH available (OR = 0.86, *p* = 0.079). The EBT investment index was not significantly associated with MST, TFC, and ACT availability.

**Table 3:** Associations between EBT fiscal and policy supports and availability of six adult and child EBTs

Outcome	Fiscal supports to promote EBT adoption			Policy supports to promote EBT adoption		
	OR	95% CI	SE	OR	95% CI	SE
Child EBTs						
Therapeutic foster care^a^	1.091	(0.954, 1.248)	0.068	1.018	(0.905, 1.145)	0.060
Multisystemic therapy^b^	1.056	(0.937, 1.190)	0.061	1.138*	(1.010, 1.282)	0.061
Functional family therapy^b^	1.132*	(1.003, 1.277)	0.061	1.176**	(1.068, 1.296)	0.049
Adult EBTs						
Supported housing^b^	0.864^	(0.734, 1.017)	0.082	0.977	(0.868, 1.101)	0.060
Supported employment^b^	1.304***	(1.148, 1.480)	0.064	1.059	(0.966, 1.162)	0.047
Assertive community treatment^b^	1.107	(0.958, 1.280)	0.073	0.992	(0.893, 1.102)	0.053
	Unstandardized coef	SE		Unstandardized coef	SE	
EBT Adoption Index^c^	0.079	0.083		0.066	0.076	

*EBT* evidence-based treatment

^a^Adjusted for linear time centered at 2002 and quadratic time centered at 2002

^b^Adjusted for linear time centered at 2002

^c^Adjusted for linear time centered at 2007

^ *p* < 0.10, * *p* < 0.05, ** *p* < 0. 01, *** *p* < 0.001

#### Policy supports and specific EBT availability

Table 3 displays the MLM estimates for the models exploring the associations between policy supports to promote EBT adoption and the availability of adult and child EBTs. Each 1 point increase in the EBT policy index was associated with an 18% increase in the odds a state will have FFT available (OR = 1.18, *p* = 0.001). Each 1 point increase in the EBT policy index was associated with a 13% increase in the odds a state will have MST available (OR = 1.13, *p* = 0.034). The EBT policy index was not significantly associated with TFC, SH, ACT, or SE availability.

## Discussion

State policy and fiscal factors are frequently observed to be influential determinants of uptake and successful implementation of EBT. Meanwhile, prominent implementation frameworks [8, 9] consistently reference the influence of the outer setting. As such, the lack of empirical examination of relationships between these “outer setting” factors and EBT investments is problematic.

The current study attempted to explore the nature of these relationships, specific to behavioral health EBT. Among 34 predictor variables examined across our proposed domains (see Fig. 1 and Table 1), only a few emerged as meaningfully related to funding and policy supports for EBT implementation. Results suggest that states’ *funding* of EBT and associated infrastructures is statistically predicted by a small number of unmodifiable state factors, including state per capita income, expansion of Medicaid under the PPACA, and Democratic political control. Two modifiable factors—presence of state behavioral health research entities (both within and outside of the SMHA) and degree of interagency collaboration—also were associated with EBT funding.

By contrast, presence of EBT-supportive *policies* was found to be predicted only by modifiable state factors. As was the case for EBT funding, interagency collaboration and presence of SMHA research entities predicted policy supports for EBT. In addition, presence of leaders from other government agencies on SMHA oversight groups and degree of independence of the SMHA from other government agencies also predicted the EBT “policy index.”

Finally, we asked how this array of state-level factors (state characteristics as well as EBT funding and policies) may influence actual EBT adoption. Only three state characteristics emerged: states where the SMHA directly operates community-based programs (as opposed to merely funding services; a modifiable factor) were found to have greater rates of EBT adoption, while Midwestern states and states with higher state budget surplus (i.e., lower debt) per capita had lower rates of EBT. Meanwhile, an interesting trend emerged whereby states’ fiscal investments in EBT (as assessed by an index of all fiscal items on the NRI survey) was associated primarily with adoption of two of the three *adult* EBTs examined (SH and SE), while states’ “EBT policy index” was associated with adoption of two of three *child* EBTs examined (MST and FFT).

### Implications for EBT implementation in mental health systems

Although the aims, methods, and data sources of this study are unique, many of its findings align with previous research. For example, Purtle and colleagues [7] found that Democratic state legislators were significantly more likely than Republicans to identify University-based research as a credible source of information. Similarly, a national scan by the Pew Charitable Trusts found that leading states in promoting evidence-based policy-making were more likely to be under Democratic than Republican control [41].

The finding that higher per capita income is associated with fiscal investments in EBT but that state budget strength was inversely related to such investments may seem counterintuitive on the surface; however, economic policy research has generally found that state-level infrastructure spending (which could extend to EBT implementation infrastructure) (1) tends to be unrelated to state per capita income [42–44]; (2) is more common among Democratically controlled states (also related to EBT investment) [44, 45]; and, not surprisingly, (3) is associated with higher per capita state debt [46]. Although discussion of these dynamics is beyond the scope of this paper, such findings about the influence of “unmodifiable state factors” on EBT implementation investment provide a starting point for discussion.

Findings also reinforce the salience of propositions embedded within certain extant implementation frameworks. For example, the EPIS framework [8] identifies “bridging factors” such as community-academic partnerships and use of purveyors and intermediary organizations as potentially important strategies for linking the outer setting to organizational (inner setting) efforts to implement EBT. As discussed below, these factors emerged in the current study as potentially critical predictors of EBT uptake. Given the increasing use of frameworks such as EPIS [18] to actively facilitate implementation efforts internationally, evidence of such concordance is encouraging.

#### Identifying potential outer setting strategies

Perhaps more important to public behavioral health stakeholders are findings regarding malleable factors that may be employed to support use of EBT and research evidence. As one example, there was a robust association between interagency collaboration and broad representation on decision-making bodies and state EBT funding and policy. Interagency collaboration or linkage (a.k.a., cosmopolitanism) is a commonly identified characteristic of the outer setting with considerable implications for implementation success [9, 47]. Research by Palinkas and colleagues [48], Hawkins and Catalano [49], and others highlight the importance of building outer setting strategies that provide “operating systems” that feature social processes that build inter-organizational linkages as strategies to promote use of research evidence.

At the same time, results suggest that support for EBT is weaker in states where SMHAs are located within an agency with broader oversight, such as a Department of Health or an omnibus agency. Taken together, these results may point to a need for policy-oriented implementation strategies that facilitate fiscal and decision-making autonomy among state behavioral health authorities, along with robust collaboration with other state agencies (e.g., Medicaid, healthcare, and human service agencies such as developmental disabilities, criminal/juvenile justice, and child welfare) and opportunities for social exchange with peers from other agencies.

Other results point to EBT support being associated with presence of research and evaluation centers—both within as well as outside of the SMHA. Although little empirical research has been done on such entities, the role of intermediary organizations that collect and mobilize fidelity, outcomes, and other data and that support use of evidence in other ways (e.g., training, consultation, convening stakeholders) has been observed to be critical to EBT adoption [50–54].

Finally, the current study uncovered an intriguing trend whereby adoption of adult-focused EBTs was associated with *fiscal investment*, while adoption of child-focused EBTs was associated with the number of EBT-supportive *policies*. One possible explanation is that children with serious behavioral health issues (and that are the focus of EBTs such as MST, FFT, and TFC) are more likely to have needs that require attention from multiple agencies than adults, even those with serious mental illness. Given that child-serving sectors such as child welfare, education, mental health, and juvenile justice all have unique missions and mandates, service reform efforts for children—including EBT adoption—will require both fiscal supports and outer setting strategies that promote collaboration, such as state and local collaborative oversight entities, interoperable data systems, and development of memoranda of agreement [55–57].

Another explanation is that, because children’s mental health problems are viewed as more transitory and less chronic and societally burdensome than problems faced by adults with serious mental illness, support for investment in children’s EBT is framed as interest in overall health promotion throughout the lifespan rather than investment in specific EBTs. Thus, attention to children’s mental health needs through EBT adoption may be more likely to occur through social processes (such as collaborative planning across agencies, cross-disciplinary training, or cross system coordination) than with targeting of fiscal resources for specific EBTs.

### Implications for further research

The potential importance of the current findings for EBT and public health—combined with the lack of previous research on outer setting drivers of EBT investment and uptake—points to the critical need for additional, more rigorous, implementation policy research. The research and funding gap is stark: while a recent review found 366 published implementation science measures, only four of these focused on the outer setting (compared to 98 for the inner setting and 90 for individuals) [21]. Meanwhile, a recent review found that policy research constituted only 10.5% of all of National Institute of Health (NIH)-funded dissemination and implementation research, which, in turn, represented less than 0.1% of all research funding [16]. The current study also highlights the likelihood that outer setting implementation determinants and strategies may differentially affect children’s services vs. adult services and thus a need to attend to these differences in future research.

This is not to say that efforts have not been undertaken. Powell and colleagues, for example, used an expert consensus process to identify 73 potential EBT implementation strategies for use in research and practice. Upwards of 32 of these strategies primarily target the outer setting [24]. Many are at the policy level and provide more complete and operationalized descriptions of implementation support activity than is included in the NRI surveys.

Efforts to extend efforts such as Powell and colleagues’ [24] to new or existing data collection efforts by federal agencies, such as the one that provided the data for the current study, would greatly improve our understanding of the outer setting of the implementation ecology. As one step, state implementation variables such as those incorporated into the NRI surveys could be cross-walked to the strategies identified by Powell et al. and/or better aligned with implementation science in other ways. In general, a more systematic approach to continually assessing state-level factors and strategies at the outer setting—along with sentinel indicators of EBT uptake and client outcomes—could be a highly productive way to expand the research base and shape more effective policy.

### Limitations

A clear limitation of the current study is the extremely limited nature of the measures of EBT implementation and the lack of any measure of impact on child outcomes. Because of the lack of consistent data across states, we were forced to rely on self-report of a small number of SMHA representatives for the majority of our independent variables as well as the dependent variable. SMHA respondents may not have been fully informed about the array of state behavioral health initiatives when responding to NRI-administered surveys. Moreover, their perceptions of the presence or absence of supports for EBT may have been colored by their perceptions of the availability of the specific EBTs (and vice-versa), or other factors, such as their time in office, role in implementation activities, clinical experience, or political affiliation. And, as with all such studies, there is a chance that the associations found in our data were upwardly biased due to response bias of participants being consistently positive or negative across items.

In the future, federal efforts to understand EBT implementation would benefit from more robust data collection methods, such as via mandates around consistent coding of EBT utilization in Medicaid and/or insurance claims. Although challenging to adopt nationally, some states have adopted such procedures [58], which could be encouraged nationally. At the very least, entities such as NRI could enact small steps toward greater rigor in its surveying methods, such as collecting information on the characteristics of reporting officials, to include in multi-level models.

Another limitation is that SMHAs are not the only systems that provide EBTs in a state. Vocational rehabilitation, child welfare, and/or juvenile justice agencies may support EBTs such as SE, TFC, MST, and others. Relatedly, we only examined relationships at a state level, when fiscal, political, policy, and other factors—as well as EBP adoption—are all likely to vary greatly within states, across jurisdictions such as counties and metropolitan areas. Another limitation is that, due to the NRI’s funding mandate to focus on services funded by federal block grants, EBT data focus only on a limited number of service types for adults with serious mental illness or children with serious emotional disturbance, not populations with other conditions or less intensive needs. The extent to which these findings are applicable to EBT implementation for other conditions, less intensive needs, or early intervention and prevention is unknown.

Given the current study capitalized on the unique federalist policy environment of the USA, it is also unclear the degree to which the current findings might apply to implementation efforts outside the USA. Nonetheless, as described above, findings from this study were found to align with implementation frameworks such as EPIS (particularly related to “bridging activities” from the outer to inner contexts that may be needed to support EBT implementation), which has been applied to implementation efforts across a range of international contexts [8].

This study is also limited in that it was exploratory and featured a large number of analyses, so is prone to familywise error. Finally, for most models reported in the tables, terms were fixed due to limited power.

## Conclusions

Despite their importance, research on the influence of outer setting factors in implementation is scarce. This study found that “unmodifiable” factors (e.g., per capita income, controlling political party) predicted funding support for mental health EBTs, while “modifiable” factors (e.g., collaboration, investment in research) predicted presence of EBT-supportive state policies. The study also found that different types of outer setting factors were associated with adoption of adult EBTs versus child EBTs.

As future research efforts yield additional insights, results can be combined with the findings from this study to provide a more complete picture of the influence of the outer setting. One might envision a near future in which we are equipped with a more complete and research-based set of “common elements” of effective policy, funding, and collaboration strategies. Such knowledge promises to promote an array of benefits, including a more useful research base, better decision-making by policy-makers, more effective surveillance and quality assurance, and more positive EBT implementation outcomes, all of which, in turn, hold promise to improve the provision of mental health services and the health of the public.

## Data Availability

The datasets generated and analyzed during the study that is described in this protocol will be available from the corresponding author on reasonable request.

## Abbreviations

ACT: Assertive community treatment
CFIR: Consolidated Framework for Implementation Research
EBT: Evidence-based treatment
FFT: Functional family therapy
HLM: Hierarchical linear modeling
MLM: Multi-level modeling
MST: Multisystemic therapy
NASMHPD: National Association of State Mental Health Program Directors
NRI: NASMHPD Research Institute
PPACA: Patient Protection and Affordable Care Act
SE: Supported employment
SH: Supported housing
SIRC: Society for Implementation Research Collaboration
SMHA: State mental health authority
SPS: State Profiles System
STATA: Syllabic abbreviation of the words statistics and data
TFC: Therapeutic foster care
URS: Uniform Reporting System

## References

[1] Bond GR, Campbell K (2008). Evidence-based practices for individuals with severe mental illness. J Rehabil.

[2] Chorpita BF, Daleiden EL (2014). Structuring the collaboration of science and service in pursuit of a shared vision. J Clin Child Adolesc Psychol.

[3] Daleiden EL, Chorpita BF (2005). From data to wisdom: quality improvement strategies supporting large-scale implementation of evidence-based services. Child Adolesc Psychiatr Clin N Am.

[4] Nakamura BJ, Chorpita BF, Hirsch M, Daleiden E, Slavin L, Amundson MJ (2011). Large-scale implementation of evidence-based treatments for children 10 years later: Hawaii’s evidence-based services initiative in children’s mental health. Clin Psychol Sci Pract.

[5] Prinz RJ, Sanders MR, Shapiro CJ, Whitaker DJ, Lutzker JR (2009). Population-based prevention of child maltreatment: the US triple P system population trial. Prev Sci.

[6] Hoagwood K, Atkins M, Ialongo N (2013). Unpacking the black box of implementation: the next generation for policy, research and practice. Adm Policy Ment Health Ment Health Serv Res.

[7] Purtle J, Dodson EA, Nelson K, Meisel ZF, Brownson RC (2018). Legislators’ sources of behavioral health research and preferences for dissemination: variations by political party. Psychiatr Serv.

[8] Aarons GA, Hurlburt M, Horwitz SM (2011). Advancing a conceptual model of evidence-based practice implementation in public service sectors. Admin Pol Ment Health.

[9] Damschroder LJ, Aron DC, Keith RE, Kirsh SR, Alexander JA, Lowery JC (2009). Fostering implementation of health services research findings into practice: a consolidated framework for advancing implementation science. Implement Sci.

[10] Watson DP, Adams EL, Shue S, Coates H, McGuire A, Chesher J, Jackson J, Omenka OI. Defining the external implementation context: an integrative systematic literature review. BMC Health Serv Res. 2018;18:209. 10.1186/s12913-018-3046-5. 10.1186/s12913-018-3046-5 PMC5870506 29580251

[11] Nilsen P, Ståhl C, Roback K, Cairney P (2013). Never the twain shall meet? - a comparison of implementation science and policy implementation research. Implement Sci.

[12] Powell BJ, Beidas RS, Rubin RM, Stewart RE, Wolk CB, Matlin SL (2016). Applying the policy ecology framework to Philadelphia’s behavioral health transformation efforts. Admin Pol Ment Health.

[13] Fisher WH, Rivard JC (2010). The research potential of administrative data from state mental health agencies. Psychiatr Serv.

[14] U.S. Department of Health and Human Services (2003). Achieving the promise: transforming mental health care in America. (DHHS Publication No. SMA-03-3832).

[15] U.S. Department of Health and Human Services (2012). 2011 medicaid managed care enrollment report.

[16] Purtle J, Peters R, Brownson RC (2016). A review of policy dissemination and implementation research funded by the National Institutes of Health, 2007–2014. Implement Sci.

[17] Frueh BC, Ford JD, Elhai JD, Grubaugh AL. (2012). Evidence-based practice in adult mental health. In: Handbook of evidence-based practice in clinical psychology. (Vol. 2 Adult disorders; I. Overview and foundational issues). Retrieved from https://onlinelibrary.wiley.com/doi/abs/10.1002/9781118156391.ebcp002001

[18] Moullin JC, Dickson KS, Stadnick NA, Rabin B, Aarons GA (2019). Systematic review of the exploration, preparation, implementation, sustainment (EPIS) framework. Implement Sci.

[19] Bruns EJ, Kerns SEU, Pullmann MD, Hensley SW, Lutterman T, Hoagwood KE (2015). Research, data, and evidence-based treatment use in state behavioral health systems, 2001–2012. Psychiatr Serv.

[20] Proctor EK, Landsverk J, Aarons G, Chambers D, Glisson C, Mittman B (2009). Implementation research in mental health services: an emerging science with conceptual, methodological, and training challenges. Adm Policy Ment Health Ment Health Serv Res.

[21] Lewis CC, Stanick CF, Martinez RG, Weiner BJ, Kim M, Barwick M (2015). The society for implementation research collaboration instrument review project: a methodology to promote rigorous evaluation. Implement Sci.

[22] Harris JR, Cheadle A, Hannon PA, Lichiello P, Forehand M, Mahoney E (2011). A framework for disseminating evidence-based health promotion practices. Prev Chronic Dis.

[23] Magnabosco JL (2006). Innovations in mental health services implementation: a report on state-level data from the U.S. evidence-based practices project. Implement Sci.

[24] Powell BJ, Waltz TJ, Chinman MJ, Damschroder LJ, Smith JL, Matthieu MM (2015). A refined compilation of implementation strategies: results from the expert recommendations for implementing change (ERIC) project. Implement Sci.

[25] U.S. Census Bureau (2016). American Community Survey 5-year estimates [Data file]*.* Retrieved from http://factfinder2.census.gov/faces

[26] U. S Department of Commerce (2016). Economic Indicators Data [Data file]*.* Retrieved from https://www.census.gov/economic-indicators/

[27] Kaiser Family Foundation (2017). Marketplace Enrollment by Congressional District, 2017 [Data file]*.* Retrieved from https://www.kff.org/interactive/interactive-maps-estimates-of-enrollment-in-aca-marketplaces-and-medicaid-expansion/

[28] Carl Klarner Dataverse (Klarnerpolitics). Harvard University, Cambridge, MA. 2013. Retrieved from https://dataverse.harvard.edu/dataverse/cklarner. Accessed 14 Mar 2019.

[29] Farmer EMZ, Burns BJ, Dubs MS, Thompson S (2002). Assessing conformity to standards for therapeutic foster care. J Emot Behav Disord.

[30] Osei GK, Gorey KM, Jozefowicz DMH (2015). Delinquency and crime prevention: overview of research comparing therapeutic foster care and group care. Child Youth Care Forum.

[31] Henggeler SW (2011). Efficacy studies to large-scale transport: the development and validation of multisystemic therapy programs. Annu Rev Clin Psychol.

[32] Henggeler SW, Schoenwald SK, Borduin CM, Rowland MD, Cunningham PB (2009). Multisystemic therapy for antisocial behavior in children and adolescents.

[33] Sexton TL, Alexander JF (2003). Functional family therapy: a mature clinical model for working with at-risk adolescents and their families. Handbook of family therapy: the science and practice of working with families and couples.

[34] Tabol C, Drebing C, Rosenheck R (2010). Studies of “supported” and “supportive” housing: a comprehensive review of model descriptions and measurement. Eval Program Plann.

[35] Cook JA, Leff HS, Blyler CR, Gold PB, Goldberg RW, Mueser KT (2005). Results of a multisite randomized trial of supported employment interventions for individuals with severe mental illness. Arch Gen Psychiatry.

[36] Drake RE, McHugo GJ, Clark RE, Teague GB, Xie H, Miles K (1998). Assertive community treatment for patients with co-occurring severe mental illness and substance use disorder. Am J Orthop.

[37] Bond Gary R., Drake Robert E. (2015). The critical ingredients of assertive community treatment. World Psychiatry.

[38] Corp S (2013). Stata statistical software (release 13) [computer software].

[39] Raudenbush SW, Bryk AS, Cheong YF, Congdon RT, du Toit M (2011). Hierarchical linear modeling (version 7.0) [computer software].

[40] Raudenbush SW, Bryk AS (2002). Hierarchical linear models: applications and data analysis methods.

[41] Davies E, Dube S, Farver M, Silloway T, Sutherland K, White D (2017). How states engage in evidence-based policymaking: a national assessment.

[42] Chernick H (2010). Redistribution at the state and local level: consequences for economic growth. Public Finance Rev.

[43] Bae S, Moon S, Jung C (2012). Economic effects of state-level tax and expenditure limitations. Public Adm Rev.

[44] Alms J, Rogers J (2011). Do state fiscal policies affect state economic growth?. Public Finance Rev.

[45] Chang C-P, Kim Y, Ying Y (2009). Economics and politics in the United States: a state-level investigation. J Econ Policy Reform.

[46] Walczak J, Malm L. (2015). Where does your state stand on state & local debt per capita? Retrieved from https://taxfoundation.org/where-does-your-state-stand-state-local-debt-capita/

[47] Aarons GA, Hurlburt M, Horwitz SM (2011). Advancing a conceptual model of evidence-based practice implementation in public service sectors. Adm Policy Ment Health Ment Health Serv Res.

[48] Palinkas LA, Fuentes D, Finno M, Garcia AR, Holloway IW, Chamberlain P (2014). Inter-organizational collaboration in the implementation of evidence-based practices among public agencies serving abused and neglected youth. Adm Policy Ment Health Ment Health Serv Res.

[49] Hawkins JD, Catalano RF, Arthur MW (2002). Promoting science-based prevention in communities. Addict Behav.

[50] Bruns EJ, Hoagwood KE, Hamilton JD (2008). State implementation of evidence-based practice for youths, part I: responses to the state of the evidence. J Am Acad Child Adolesc Psychiatry.

[51] Bruns EJ, Hoagwood KE, Rivard JC, Wotring J, Marsenich L, Carter B (2008). State implementation of evidence-based practice for youths, part II: recommendations for research and policy. J Am Acad Child Adolesc Psychiatry.

[52] Proctor E, Hooley C, Morse A, McCrary S, Kim H, Kohl P (2019). Intermediary/purveyor organizations for evidence-based interventions in the US child mental health: characteristics and implementation strategies. Impliment Sci.

[53] Fixsen DL, Naoom SF, Blase KA, Friedman RM, Wallace F (2005). Implementation research: a synthesis of the literature.

[54] Franks RP, Bory CT (2015). Who supports the successful implementation and sustainability of evidence-based practices? Defining and understanding the roles of intermediary and purveyor organizations. New Dir Child Adolesc Dev.

[55] Bruns EJ, Walker JS, Bernstein A, Daleiden E, Pullmann MD, Chorpita BF (2014). Family voice with informed choice: coordinating wraparound with research-based treatment for children and adolescents. J Clin Child Adolesc Psychol.

[56] Friedman RM, Drews DA (2005). Evidence-based practices, systems of care, & individualized care.

[57] Walker JS, Sanders B (2011). The community supports for wraparound inventory: an assessment of the implementation context for wraparound. J Child Fam Stud.

[58] Walker SC, Sedlar G, Berliner L, Rodriguez FI, Davis PA, Johnson S, Leith J. Advancing the state-level tracking of evidence-based practices: a case study. Int J Ment Heal Syst. 2019;13(25). 10.1186/s13033-019-0280-0. 10.1186/s13033-019-0280-0 PMC6457070 31007712

